# Cerebral Autoregulation: A Target for Improving Neurological Outcomes in Extracorporeal Life Support

**DOI:** 10.1007/s12028-024-02002-5

**Published:** 2024-05-29

**Authors:** Nolan Chalifoux, Tiffany Ko, Julia Slovis, Audrey Spelde, Todd Kilbaugh, Constantine D. Mavroudis

**Affiliations:** 1 Department of Clinical Sciences and Advanced Medicine, School of Veterinary Medicine, University of Pennsylvania, Philadelphia, PA 19104 USA; 2 Department of Anesthesiology and Critical Care Medicine, University of Pennsylvania, Philadelphia, PA 19104 USA; 3 Division of Cardiothoracic Surgery, Children’s Hospital of Philadelphia, Philadelphia, PA 19104 USA; 4 Institute for Translational Medicine and Therapeutics, Perelman School of Medicine, University of Pennsylvania, Philadelphia, PA 19104 USA

**Keywords:** Extracorporeal membrane oxygenation, Cerebrovascular circulation, Intracranial pressure, Cerebral hemorrhage, Stroke, Cardiopulmonary resuscitation

## Abstract

Despite improvements in survival after illnesses requiring extracorporeal life support, cerebral injury continues to hinder successful outcomes. Cerebral autoregulation (CA) is an innate protective mechanism that maintains constant cerebral blood flow in the face of varying systemic blood pressure. However, it is impaired in certain disease states and, potentially, following initiation of extracorporeal circulatory support. In this review, we first discuss patient-related factors pertaining to venovenous and venoarterial extracorporeal membrane oxygenation (ECMO) and their potential role in CA impairment. Next, we examine factors intrinsic to ECMO that may affect CA, such as cannulation, changes in pulsatility, the inflammatory and adaptive immune response, intracranial hemorrhage, and ischemic stroke, in addition to ECMO management factors, such as oxygenation, ventilation, flow rates, and blood pressure management. We highlight potential mechanisms that lead to disruption of CA in both pediatric and adult populations, the challenges of measuring CA in these patients, and potential associations with neurological outcome. Altogether, we discuss individualized CA monitoring as a potential target for improving neurological outcomes in extracorporeal life support.

## Introduction

Despite improvements in survival after illnesses requiring extracorporeal life support (ECLS), neurologic injury remains a persistent and occasionally devastating complication [1–4]. As the use of ECLS continues to increase for cardiopulmonary failure [5–8], there is an ever-growing necessity to understand factors that contribute to such neurologic injury. Identification of these factors is key to preventing injury and to developing potential targets for therapeutic interventions via patient-directed physiologic targets and molecular therapeutics. One such target is alterations in cerebral autoregulation (CA). The cerebral vasculature uses autoregulation as an innate protective mechanism, a concept first elucidated by Fog in 1930 when vasodilation of the pial artery was observed in response to experimental hypotension [9]. This mechanism uses both mechanical and chemical factors to maintain constant cerebral blood flow (CBF) across a range of systemic blood pressures [10]. This vasomotor adaptability is impaired in certain disease states and potentially by ECLS itself [11, 12]. Patients requiring extracorporeal membrane oxygenation (ECMO) are at significant risk of cerebral injury [13], and dysregulation of CA may play a primary role, but our knowledge of ECMO’s impact on CA remains limited. Therefore, there is a need to further understand the pathophysiology of how extracorporeal circulation affects the vasomotor reactivity of the cerebral vasculature and its potential clinical implications in the prevention of neurological injury.

**Fig. 1:**
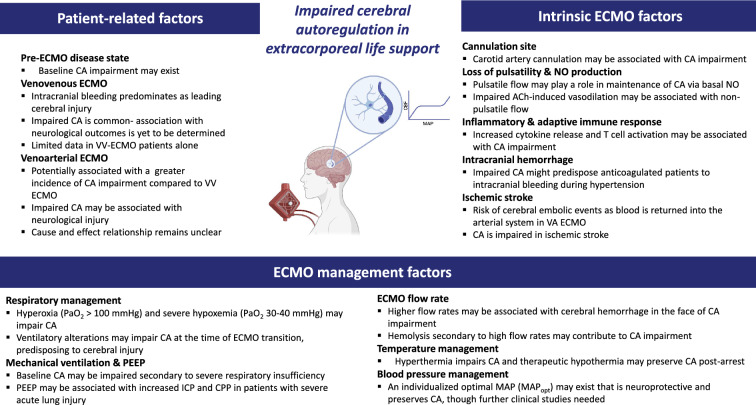
Summary of potential factors contributing to CA disruption in extracorporeal life support. Created with BioRender.com. *Ach* acetylcholine, *CA* cerebral autoregulation, *CBF* cerebral blood flow, *CPP* cerebral perfusion pressure, *ECMO* extracorporeal membrane oxygenation, *ICP* intracranial pressure, *MAP* mean arterial pressure, *NO* nitric oxide, *PaO*_*2*_, partial pressure of oxygen; *PEEP* positive end-expiratory pressure, *VA* venoarterial, *VV* venovenous

The aim of this narrative review is to discuss the physiology of CA and to highlight current understanding of potential mechanisms that lead to disruption of CA during states of critical illness in which venovenous extracorporeal membrane oxygenation (VV ECMO) and venoarterial extracorporeal membrane oxygenation (VA ECMO) are employed. We highlight the challenges of measuring CA in these patients as well as available evidence to date regarding the association of CA with neurological outcomes (Fig. 1).

## Physiology and Monitoring of CA

### Physiology

Cerebral autoregulation is defined as the cerebral regulatory mechanism that maintains a constant CBF over wide ranges of cerebral perfusion pressures (CPPs) and, by association, arterial blood pressures [9, 10]. CA maintains a constant CBF by four different mechanisms: myogenic, metabolic, endothelial, and neurogenic. The myogenic mechanism is characterized by changes in the smooth muscle tone of small arteries and arterioles in response to changes in hydrostatic pressure [10]. In contrast, the metabolic mechanism is characterized by changes of the microvasculature tone in response to vasoactive stimuli within the microenvironment, such as pH, the partial pressure of carbon dioxide and the partial pressure of oxygen [14]. The third mechanism originates from the vascular endothelium and involves the paracrine secretion of vasoactive substances, such as nitric oxide (NO) (a vasodilator) and endothelin-1 and thromboxane A2 (vasoconstrictors) [15]. Proposed triggers include shear stress and changes in transmural pressure [16]. The neurogenic mechanism remains to be fully elucidated; however, it is hypothesized to involve the secretion of various vasoactive neurotransmitters by neuroglial cells innervating cerebral vessels [17]. It has been traditionally believed that under normal circumstances, these mechanisms collaborate to prevent cerebral injury over a range of mean arterial pressures (MAPs) that fall between a presumptive lower limit of autoregulation (LLA) and an upper limit of autoregulation (ULA); this range is commonly reported to lie between 50 and 150 mm Hg in adult patients [18]. A MAP below the LLA (e.g., 50 mm Hg) increases the risk of cerebral ischemia; comparatively, a MAP above the ULA (e.g., 150 mm Hg) increases the risk of cerebral hyperemia [18–20]. However, contemporary data challenges the traditional dogmatic view of CA and suggests that the CA plateau, should it truly exist, is far narrower than traditionally reported and is individual specific [21, 22]. Furthermore, changes in CBF remain highly susceptible to various physiological and clinical alterations, such as physical manipulations (i.e., head-up tilt) and vasoactive drugs [21]. The protective response of the cerebral vasculature is now thought to be dependent on not only the speed at which MAP changes but also the directionality of the change [21, 22]. Alterations in the limits and mechanisms of CA in neonates and infants remain incompletely understood.

### Monitoring of CA

Various methods of CA monitoring have been reported (see Table 1). Generally, concurrent samples of CBF (or a surrogate) and CPP (or a surrogate) over time are correlated to generate an index of CA. Because CPP is defined as the difference between MAP (a clinically accessible parameter) and the critical closing pressure (intracranial pressure [ICP] + vessel wall tension), MAP is frequently substituted for CPP for CA monitoring. However, it is important to note that a subset of patients may have increased ICP secondary to an underlying disease process and that MAP is not a universally appropriate surrogate. When CA is intact, a negative or near-zero correlation is expected as cerebral neuroprotective mechanisms work to maintain constant CBF irrespective of any shift in MAP. As CA is impaired, CBF and MAP become more positively correlated and CA indices trend toward 1. The low and high values of MAP in which the CA index surpasses its cutoff, indicating CA disruption, may be used to define the LLA and ULA, respectively. A numerically determined nadir of the U-shape relationship between the CA index and MAP is commonly used to define an optimal MAP (MAP_opt_) in which CA is intact.

**Table 1:** Summary of population, measurement of CA, neurological assessment, and key findings of experimental and clinical studies reporting on CA in extracorporeal life support

	Population	Year	*N*	Indication	CA measurement	Ref	Key findings
VV ECMO	Newborn lambs (1–7 days old)	1996	6 VV, 6 controls (ligation of right jugular vein without ECMO)	Experimental	Lower limit of autoregulation	[68]	At a CPP < 25 mm Hg, CBF, cerebral oxygen delivery, and metabolic rate were lower in the ECMO group than in controls, and fractional oxygen extraction and cerebral vascular resistance were higher, suggesting impaired CA
	Adults (> 18 years old)	2021	66 total; 19 required VV ECMO	ARDS (acute phase)	COx between NIRS-derived rSO_2_ and MAP	[33]	No significant association between early hypercapnia and impaired CA; hypocapnia during the monitoring period was significantly associated with impaired CA. Percentage of time with impaired CA ranged from 21.9% ± 16.9% to 28.1% ± 13.7% for patients with acute brain injury Neurological assessment: acute brain injury on neuroimaging during ICU stay, new central nervous system disorder, and functional outcome at 3 months
VA ECMO	Pediatrics	1987	13 total; 13 VA	Severe respiratory failure	Correlation between TCD-derived CBF and MAP	[51]	With the onset of ECMO, CBF appears to increase (marked increases in the area under the velocity curve) in association with increasing MAP and PaCO_2_ levels, suggesting impaired CA
	Newborn lambs (1–7 days)	1993	7 VA, 7 controls (ligation of right carotid artery and jugular vein without ECMO)	Experimental	Lower limit of autoregulation	[70]	CBF and cerebral oxygen consumption decreased from baseline in ECMO cohort at CPP of 39–25 and < 25 mm Hg verus remained unchanged in controls until CPP < 25 mm Hg. When CA appeared altered, right-left hemispheric CBF differences were noted in both groups
	Pediatrics (1–25 days)	2012	6 VA (5 neonatal respiratory failure, 1 post cardiac surgery)	Neonatal respiratory failure (5), post cardiac surgery (1)	WCC between NIRS-derived HbO_2_ and MAP	[49]	WCC increases with decreasing ECMO flow, demonstrating loss of CA at low ECMO flows. Significant differences in WCC were observed between the right and left scalp, with channels on the right exhibiting higher values of WCC, suggesting that the right hemisphere was more susceptible to CA impairment
	Pediatrics (1–6 days old)	2020	9 total VA	PPHN (1), congenital diaphragmatic hernia (4), gastroschisis (1), tetralogy of Fallot (1), tetralogy of Fallot and PPHN (1), biventricular dysfunction (1)	Correlation coefficient (DCSx) between DCS-derived relative CBF and MAP	[42]	Average MAP and pump flow levels were weakly correlated with CBF and were not correlated with rSO_2_. CA integrity varied between individuals and with time. Systemic measurements of MAP, pulse pressure, and left cardiac dysfunction were not predictive of impaired CA
ECPR	Swine (27–35 kg)	2021	12 total ECPR (6 high MAP target, 6 standard MAP target)	Experimental	PRx between invasive ICP gauge and MAP	[140]	Target MAP 80–90 mm Hg associated with improved CA in early ECPR but associated with impaired CA post ROSC compared to standard MAP group of 65–75 mm Hg
	Pediatrics (≤ 18 years old)	2021	10 cardiac arrest patients required ECMO; 26 did not		HVx between NIRS-derived rTHb and MAP	[50]	The area under the curve for time spent with MAP below MAP_opt_ was not associated with change in the Pediatric Cerebral Performance Category score for children who required ECMO Neurological assessment: Pediatric Cerebral Performance Category score
VV and VA ECMO	Pediatrics (unspecified)	2017	25 total (11 neonatal, 14 pediatrics); combined VV and VA ECMO		WTC between NIRS-derived rSO_2_ and MAP	[11]	Significant correlations between individual CA indices and neuroimaging scores were found in both neonates and children. Higher MAP variability was associated with impaired CA in children but not neonates Neurological assessment: neuroimaging score (not specified)
	Pediatrics (0–15 years old)	2017	25 total; 9 VV, 12 VA, 4 VV to VA	PPHN (10; 2 VV, 8 VA), septic shock (4; 1 VV to VA, 3 VA), ARDS (10; 7 VV, 2 VV to VA, 1 VA), septic shock + ARDS (1, VV to VA)	WTC between NIRS-derived rSO_2_ and MAP	[46]	The degree of CA impairment correlated significantly with the neuroimaging scores Neurological assessment: neuroimaging score (CT or MRI): bleeding, parenchymal lesions (ischemia or infarction), and ventricular dilatation
	Pediatrics (0–14 years old)	2019	20 total; 5 VV, 15 VA (5 Pediatric Logistic Organ Dysfunction score matched controls)		WTC between NIRS-derived rSO_2_ and MAP	[47]	Significant correlation between adaptive immune responses of ECMO patients with acquired brain injury (*n* = 9) and loss of CA Neurological assessment: acquired brain injury assessed via MRI
	Pediatrics (0–15 years old)	2020	47 total; 18 VV, 29 VA	PPHN (19), ARDS (17), septic shock (9), other (2)	WTC between NIRS-derived rSO_2_ and MAP	[48]	No-injury patients (*n* = 23) had minimal variability in CA over a broad range of MAP. The CA index had a moderate correlation with the neuroimaging scores (*R* = 0.51). Ischemic group showed significant abnormalities in CA in the lower MAP range (particularly among VA ECMO). Hemorrhagic group had highest average MAP as well as the lowest rSO_2_, suggesting elevated cerebral vascular resistance Neurological assessment: neuroimaging classification (CT or MRI): no injury, ischemic injury, and hemorrhagic injury
	Pediatrics (0–15 years old)	2021	31 total runs; 3 VV, 28 VA	Hemodynamic (24), respiratory (7)	COx between NIRS-derived rSO_2_ and MAP	[34]	Highest COx values observed on day 1 of ECMO. COx values tended to be lower in case of hypercapnia compared to normocapnia. A weak but significant correlation between PaCO_2_ and upper limit of CA was observed (*R* = 0.432), strongest on day 1. A positive correlation between lower limit of CA and PaCO_2_ was also found on day 1 (*R* = 0.726). A very weak negative correlation between PaO_2_ and upper limit of CA was observed within the whole ECMO run (*R* = − 0.07) and on day 1 (*R* = − 0.135). The span of the CA plateau was positively correlated with PaCO_2_ (R = 0.224) and negatively correlated with PaO_2_ (*R* = − 0.051)
VV ECMO, VA ECMO, and ECPR	Pediatrics (0–15 years old)	2021	29 total; 7 VV, 21 VA, 1 ECPR	Hemodynamic (18), respiratory (10), ECPR (1)	COx between NIRS-derived rSO_2_ and MAP	[35]	Mean COx was higher during day 1 of ECMO compared to day 2. Mean COx and percentage of time spent with a COx > 0.3 were significantly higher among patients with an acute neurological event. These patients spent significantly more time with MAP below the lower limit and above the upper limit of CA Neurological assessment: acute neurological event defined as hemorrhagic or ischemic stroke and/or clinical or electrical seizure and/or brain death
	Adults (21–78 years)	2022	13 total; 2 VV, 4 VA, 7 VA for cardiac arrest	ARDS (2; 2 VV), acute heart failure (4; 4 VA), cardiac arrest (7; 7 VA)	CBF asymmetry via TCD and DCS-derived relative CBF (left and right hemispheres)	[52]	Comatose patients (*n* = 4; 1 VA, 3 VA for cardiac arrest) had greater interhemispheric regional blood flow asymmetry versus noncomatose patients over a range of MAP values. Regional blood flow asymmetry in comatose patients resolved near a MAP range of 70–80 mm Hg, whereas blood flow remained symmetric through a wider MAP range in noncomatose patients Neurological assessment: comatose (Glasgow Coma Scale motor score ≤ 4) versus noncomatose patients (Glasgow Coma Scale motor score > 4)

*ARDS* acute respiratory distress syndrome, *CA* cerebral autoregulation, *CBF* cerebral blood flow, *COx* cerebral oximetry index, *CPP* cerebral perfusion pressure, *CT* computed tomography, *ECMO* extracorporeal membrane oxygenation, *ECPR* extracorporeal cardiopulmonary resuscitation, *DCS* diffuse correlation spectroscopy, *DCSx* diffuse correlation spectroscopy index, *HbO*_*2*_ oxyhemoglobin concentration, *HVx* hemoglobin volume index, *ICP* intracranial pressure, *ICU* intensive care unit, *MAP* mean arterial pressure, *MAP*_*opt*_ optimal mean arterial pressure, *MRI* magnetic resonance imaging, *NIRS* near-infrared spectroscopy, *PaCO*_2_ arterial partial pressure of carbon dioxide, *PaO*_2_ arterial partial pressure of oxygen, *PPHN* persistent pulmonary hypertension of the newborn, *PRx* pressure reactivity index, *ROSC* return of spontaneous circulation, *rSO*_*2*_ regional cerebral oxygen saturation, *rTHb* relative tissue hemoglobin density, *TCD* transcranial Doppler, *VA* venoarterial, *VV* venovenous, *WCC* wavelet cross-correlation, *WTC* wavelet transform coherence

The gold standard measurement of continuous CBF in humans relies on a direct measurement of radiolabeled water (^15^O-water) via positive emission tomography [23]. However, this technique is invasive and is associated with potential complications [24]. CBF surrogates available for clinical use include cerebral blood flow velocity, regional cerebral oxygen saturation (rSO_2_), tissue oxygen partial pressure, ICP, and microdialysis-derived glutamate.

Cerebral blood flow velocity is measured noninvasively via transcranial Doppler (TCD) assessment of the middle cerebral arteries. This CBF velocity is subsequently correlated with MAP to derive a flow velocity index [25–28]. TCD is a frequently used method of CA assessment [29, 30] but requires a trained technician and assumes that there is minimal change in the diameter of the middle cerebral artery during MAP fluctuations [31].

Use of noninvasive near-infrared spectroscopy (NIRS) provides two potential CBF surrogates: the rSO_2_ and an index of tissue hemoglobin concentration. rSO_2_ is correlated with MAP to derive the cerebral oximetry index (COx; cutoff for CA disruption, COx > 0.3) [32–35]. rSO_2_ terminology varies based on device and has alternatively been reported as the tissue oxygen index used to calculate a corresponding CA index (cutoff > 0.1) [25]. Similarly, measurements of tissue hemoglobin concentration are reported as total hemoglobin volume or tissue hemoglobin. These measurements are used to derive a hemoglobin volume index (cutoff > 0.3) [36] or tissue hemoglobin index (cutoff not defined) when correlated with MAP [37]. Although these measures of tissue oxygenation and metabolism provide a surrogate for CBF, it is important to consider that they may be influenced by factors other than CBF, such as mitochondrial dysfunction and oxygen diffusion barriers [38].

Variation in cutoffs for CA disruption is a reflection of not only different devices but also variable study outcomes. Thus, clinical NIRS cerebral oximetry provides noninvasive, continuous, real-time measurements that appear sensitive to cerebral hemodynamic changes; however, accuracy and reproducibility are limited by factors such as agitation and skin conditions, sensitivity to ambient lighting, limits of detection, and extracerebral contamination [39, 40]. Investigational NIRS devices are working to address these limitations through the use of frequency-domain diffuse optical spectroscopy and diffuse correlation spectroscopy (DCS) techniques, which may be combined into a single neurometabolic optical monitoring device [41, 42]. These devices permit concurrent noninvasive measure of oxyhemoglobin, deoxyhemoglobin, and total hemoglobin concentrations in tissue, rSO_2_, relative CBF, and ICP, each of which may be correlated with MAP to derive additional indices of CA. These developments show promise as an approach to real-time monitoring of CA [43–45].

Various methods have been used to measure CA in ECMO patients. These methods include NIRS-derived rSO_2_ [11, 33–35, 46–48], oxyhemoglobin concentration [49], and total hemoglobin concentration [50], in addition to TCD-derived [51] and DCS-derived [42, 52] CBF. Of note, each of these methods is used to derive a unique CA index, none of which is a gold standard. Furthermore, data evaluating CA require time-averaging over hours and are predominantly derived from experimental single-center studies in which CA indices lack validation studies and prospective investigations [22]. These remain significant limitations in the monitoring of CA. The reader is directed elsewhere for a more comprehensive review of CA monitoring technologies [37, 53–56].

### Patient-Related Factors

Prior to ECMO cannulation, it is important to consider that many patient-related factors may impact CA at baseline prior to extracorporeal intervention. Because these critically ill patients require treatment for severe disease, it is possible that the disease itself may contribute to CA impairment regardless of subsequent intervention. CA impairment has been well documented not only in conditions associated with direct cerebral injury, such as acute subarachnoid hemorrhage [57], traumatic brain injury [58], acute stroke [59], and cardiac arrest [60], but also in numerous non-brain-injured conditions [61], such as sepsis and septic shock [62] and acute respiratory distress syndrome [33]. Consequently, the pre-ECMO state is key when considering the subsequent impact of ECMO transition and its potential role in CA impairment.

### VV ECMO

Venovenous extracorporeal membrane oxygenation is commonly activated following identification of refractory pulmonary failure. The VV ECMO circuit is connected in series to the heart and lungs, providing extracorporeal respiratory support. However, VV ECMO has been associated with multiple neurological complications that are associated with increased morbidity and mortality [63–65] and occur frequently, with an incidence of 12–44% in both pediatric and adult populations [35, 47, 48, 63, 64, 66, 67]. There is increasing evidence that CA is significantly altered during VV ECMO [11, 33–35, 46–48, 68]. Consequently, this alteration may contribute to neurological injury.

Considering the numerous factors threatening the integrity of CA in patients dependent on VV ECMO, it is not unexpected that experimental and clinical evidence supports CA impairment (see Table 1). Walker et al. first documented this impairment in 1996 during VV ECMO in a model of newborn lambs [68]. Recently, studies have documented impaired CA in mixed ECMO populations as measured by NIRS [11, 34, 46–48]. However, this evidence is primarily limited to neonatal and pediatric populations with inclusion of VA ECMO patients. Kahl et al. recently described increased CA impairment in three patients and decreased CA impairment in four patients with acute respiratory distress syndrome on VV ECMO [33]. These low numbers and variability in techniques to determine CA highlight the need for more precise noninvasive bedside modalities to monitor CA.

### Association of CA with Neurological Outcome in VV ECMO

Although CA impairment is common in patients on VV ECMO, the association with neurologic outcomes has yet to be determined. Intracranial bleeding predominates as the leading cerebral injury in VV ECMO [35, 48, 63–66]. Impaired CA in cases of intracerebral hemorrhage is associated with worse neurological outcomes, such as hematoma volume and Glasgow Coma Scale score [69]; however, to date, no studies have evaluated this association in VV ECMO specifically. Examining the association between CA impairment and neurological injury in VV ECMO is needed.

### VA ECMO

The use of VA ECMO offers a life-sustaining recovery period in the face of severe cardiopulmonary failure. The VA ECMO circuit is connected in parallel to the heart and lungs, providing both respiratory and cardiovascular support. Early experimental studies in newborn lambs identified greater decreases in CBF in VA ECMO compared to VV ECMO, with a similar trend in the degree of CA impairment [68, 70]. This disproportionate neurological injury burden has been echoed among clinical studies in pediatrics and adults in which VA ECMO has been associated with an increased incidence of neurological complications, ranging from 15 to 75% [3, 35, 48, 64, 67, 71–75], compared to VV ECMO [2, 64, 66, 76, 77]. However, this comparison is limited by interstudy variability in the definition of neurological complications. Nonetheless, numerous factors unique to VA ECMO raise concern for its potential impact on CA and subsequent neurological injury.

### Association of CA with Neurological Outcome in VA ECMO

There is an abundance of evidence documenting impaired CA in VA ECMO [11, 42, 46–49, 51, 70] (Table 1). However, whether impaired CA independently causes poor neurological outcomes or is merely a consequence of cerebral injury remains poorly understood, and it is important to consider the patient’s precannulation state when assessing determinants of outcome. This is particularly true in the realm of extracorporeal cardiopulmonary resuscitation (ECPR), in which hypoxic-ischemic injury contributes significantly to neurological injury prior to the initiation of ECMO. In a pediatric population of predominantly VA ECMO patients, the percentage of time spent with impaired CA (i.e., COx > 0.3) was significantly higher among patients who had hemorrhagic or ischemic stroke, clinical or electrical seizure, and/or brain death [35]. Impaired CA has also been associated with an increasing severity of computed tomography and magnetic resonance imaging neuroimaging scores in two pediatric studies in which most patients were supported by VA ECMO [46, 48].

### Association of CA with Neurological Outcome in Combined VV and VA ECMO Populations

There is a correlation between CA impairment, as determined by a positive COx, and the severity of neuroimaging findings concerning for neurological injury in pediatric patients on VV or VA ECMO [46–48]. Furthermore, a greater percentage of time spent with an impaired CA index (COx) has been associated with the incidence of stroke, seizures, or brain death in a similar population [35]. Of particular importance, this CA impairment had been apparent prior to clinically detectable neuroimaging changes on computed tomography or magnetic resonance imaging [46]. Though further research is needed to completely elucidate the relationship between impaired CA and poor neurological outcomes, this evidence suggests that maintaining CA is critical for neuroprotection and the mitigation of neurological injury.

## Intrinsic ECMO Factors

### Cannulation Site

There are potential implications of the cannulation site and its mechanical effects on CBF, particularly for VA ECMO. Neck cannulation is frequently used in pediatrics because the femoral vessels used in adults are typically of insufficient size to allow for full support [78, 79]; however, VA ECMO neck cannulation is concerning because it involves the right internal jugular vein and the right carotid artery. Although this site has the advantage of being expeditious and avoids the risk of limb ischemia, the right cerebral hemisphere may be at increased risk of ischemic injury as it becomes dependent on collateral flow via the circle of Willis [42, 78, 79].

Following cannulation of the right carotid artery, a brief period of right unilateral cerebral tissue oxygen desaturation occurs [80]. Early studies identified an association between the incidence of right-sided cerebral injury and carotid artery cannulation [81–84]. However, asymmetric cerebral perfusion has also been reported in a mixed population of adults on both VV and VA ECMO, none of whom underwent carotid artery cannulation [52]. The role of carotid artery cannulation in lateralized cerebral damage and neurological injury remains to be elucidated, as many other investigations have failed to identify this association [66, 75, 77, 85–91].

Cerebral autoregulation impairment has been proposed to play a role in cerebral perfusion changes following neck cannulation for VA ECMO. Matsumoto et al. proposed that intact CA may have been responsible for the preservation of right middle cerebral artery velocities during ligation of the right carotid artery in hypoxic infants transitioning to VA ECMO [92]. Subsequently, Papademetriou et al. found that carotid artery ligation increased CA impairment in the ipsilateral right hemisphere but not the left hemisphere in neonates undergoing VA ECMO [49]. CA disruption has also been associated with lower ipsilateral CBF following carotid artery ligation in newborn lambs [70]. However, impaired CA has also been associated with asymmetric CBF in adults undergoing VA ECMO, none of whom had carotid artery cannulation [52]. Therefore, it is possible that impaired CA in the face of variable systemic blood pressure may play a more causative role in asymmetric perfusion and subsequent neurological injuries than cannulation site alone.

Peripheral bifemoral cannulation in adults on VA ECMO has been associated with the ejection of anterograde deoxygenated blood from the left side of the heart to the aorta, hindering the retrograde oxygenated blood as it enters the aortic arch from the extracorporeal circuit [93]. This phenomenon, known as Harlequin syndrome, may result in asymmetrical cerebral perfusion and subsequent hypoxia [93, 94]. As such, it is crucial to ensure proper ventricular unloading and to monitor right radial arterial oxygen saturations to avoid potential coronary and cerebral ischemia, which may have secondary consequences on CA.

### Loss of Pulsatility and NO Production

Pulsatile flow may play a role in the maintenance of CA. As VA ECMO partially bypasses the failing cardiovascular system, it is associated with dampened or nonpulsatile blood flow. This dampened pulsatility has been associated with an earlier impairment and larger decrease in CBF and cerebral oxygen consumption compared to VV ECMO, in which the functioning heart maintains pulsatile flow [68, 70, 95].

The importance of pulsatile blood flow in CA has been thought to be mediated through its impact on basal NO production. Acetylcholine-induced vasodilation is mediated by the endogenous release of NO by the cerebral arterial endothelium. Experimental animal models have documented an impaired response to this acetylcholine-induced vasodilation in the face of nonpulsatile cardiopulmonary bypass or VA ECMO [96–99]. As the endothelial mechanism underlying CA depends on the paracrine secretion of these vasoactive substances, impairment in this response may contribute to impaired cerebral arterial tone and CA in the face of nonpulsatile blood flow. Furthermore, recovery of the myogenic mechanism of CA has been noted following the addition of an NO donor in experimental nonpulsatile ECMO models [100].

### Inflammatory and Adaptive Immune Response

Patients transitioned to ECMO frequently have an inflammatory reaction as their blood contacts the extracorporeal circuit [101]. This inflammatory cascade may result in neutrophil activation, hypercoagulability, and organ injury [101]. Upregulation of proinflammatory cytokines and proteins has been documented in the cerebral tissues of swine following 24 h of VV ECMO [102], indicating potential for cerebral-specific injury and CA impairment.

The adaptive immune response has also been implicated as a potential contributor to impaired CA and injury in ECMO [47]. In a pediatric study of predominantly VA ECMO patients, the percentage of activated T helper cells was found to correlate with CA impairment, measured by an NIRS-derived index (COx), throughout the ECMO period in patients with cerebral injury on magnetic resonance imaging [47]. These patients had a tenfold increase in interleukin-8, a cytokine that promotes adherence of leukocytes to endothelial cells. As CA is disrupted, states of low CBF may increase leukocyte diapedesis into the cerebral parenchyma, promoting neurological injury [47].

### Intracranial Hemorrhage

Loss of CA following transition to ECMO may contribute to intracranial bleeding while the patient is anticoagulated due to passive increases in CBF with increases in MAP [103, 104]. In a pediatric mixed ECMO population, increased time spent in a higher blood pressure range (> 100% normal) was associated with the development of intracranial bleeding [48]. Similarly, time spent above ULA has also been associated with an increased risk of neurological injury, defined as hemorrhagic or ischemic stroke, seizures, or brain death [35]. However, the relationship between intact CA and intracranial hemorrhage specifically remains unclear; using the MAP and NIRS-derived rSO_2_ index of CA (COx), CA was observed to be more intact in the high blood pressure range (140% normal) versus those in the low blood pressure range for pediatric ECMO patients who developed intracranial hemorrhage [48]. Although it remains plausible that impaired CA might predispose anticoagulated patients to intracranial bleeding during more severe hypertension, intracranial bleeding itself has also been shown to impair CA [69]. Furthermore, hemorrhagic conversion of ischemic strokes may arise independent of anticoagulation, ECMO intervention, and impaired CA. Hence, there is likely an interplay between cause and effect when considering the relationship between impaired CA and intracranial bleeding.

### Ischemic Stroke

Ischemic stroke is of particular concern in VA ECMO because the circuit returns oxygenated blood directly into the arterial system, increasing the risk of cerebral embolic events [3, 35, 48, 64, 74, 84]. Additionally, thrombi originating from cardiac chambers at the early phase of myocardial infarction can also lead to acute ischemic stroke [105]. Mateen et al. identified ischemic stroke or hypoxemic-ischemic encephalopathy in 90% of adults treated with VA ECMO at autopsy [72]. CA is known to be impaired in ischemic stroke, and its progressive disruption has been associated with both cerebral edema and hemorrhagic transformation [106, 107]. Impaired CA has also been documented in pediatric VA ECMO patients with cerebral ischemic stroke [48].

## ECMO Management Factors

### Respiratory Management

#### Oxygen Delivery

It has been proposed that hyperoxia (partial pressure of oxygen [PaO_2_] > 100 mm Hg) has the potential to affect CA through platelet dysfunction, abnormal hemostasis, and oxidative stress [34, 108]. Comparatively, animal models of neonatal respiratory failure have shown that even short durations of severe hypoxemia (PaO_2_ = 30–40 mm Hg) also impair CA [109–111]. There is increased risk of cerebral hyperemia and intracranial hemorrhage at the time of transition to ECMO when rapid increases in MAP may overwhelm the dysfunctional autoregulatory system [66].

In adults, a higher PaO_2_ increase at ECMO transition has been associated with cerebral bleeding after VV ECMO onset [63]. A recent retrospective analysis found a weak negative correlation between PaO_2_ and the ULA in children treated with either VA or VV ECMO [34]. Increases in PaO_2_ resulted in decreases in the ULA, a finding that may have been driven by a greater positive delta PaO_2_ in hypoxic patients [34]. Altogether, these findings suggest that hypoxemic patients may have pressure-dependent CBF following transition to ECMO, putting them at risk of developing cerebral ischemia or hyperemia as MAP fluctuates. Alternatively, hyperoxia may also contribute to CA disruption following transition to ECLS.

#### Carbon Dioxide Maintenance

Evidence of the specific effects of hypercapnia (partial pressure of carbon dioxide [PaCO_2_] > 45 mm Hg) and hypocapnia (PaCO_2_ < 35 mm Hg) on CA are emerging but remain to be fully elucidated. Studies investigating the impact of hypercapnia on the limits of CA have identified an increase in the LLA thought to be mediated by hypercapnia-induced cerebral vasodilation [34, 112, 113]. Comparatively, the impact of hypercapnia on the ULA remains inconsistent, suggesting a lesser role in the pathogenesis of cerebral hyperemia [34, 112, 113]. Hypocapnia does not appear to appreciably change the LLA but has an ambiguous impact on the ULA [113].

Rapid correction of severe hypercapnia following transition to ECMO is associated with an increased incidence of neurological complications, in particular cerebral hemorrhage and ischemia [63, 76, 114]. However, a recent study including 29% of patients on ECMO found no association between early hypercapnia and disturbances in CA as measured by an NIRS-derived index (COx) in acute respiratory distress syndrome [33]. Comparatively, hypercapnia was found to have a potential protective effect on CA in pediatric patients on VV or VA ECMO [34]. COx tended to be lower during hypercapnia compared to normocapnia, supporting intact CA in this study [34]. Tian et al. investigated the relationship between CA and absolute PaCO_2_ change. They failed to find an association with the difference in 24-h maximum pre-ECMO values and minimum PaCO_2_ in the first 24 h on either VV or VA ECMO in pediatric patients [46].

The presence of hypocapnia prior to ECMO initiation may also be a potential risk factor of cerebral injury following transition to ECMO. Taylor et al. proposed that infants with pre-ECMO hypocapnia might be at risk of rapid absolute PaCO_2_ increases following transition to VA ECMO [95]. This increase in PaCO_2_ may result in arteriolar vasodilation and increased CBF [95]. This was further supported in a similar population in which CBF as measured by TCD was positively associated with MAP in the face of increasing PaCO_2_ at the onset of ECMO [51]. Most recently, hypocapnic episodes were found to be associated with impaired CA in adults with acute respiratory distress syndrome, among whom approximately one quarter of patients were dependent on ECMO [33].

Despite mixed results, it is likely that both the speed and amplitude of PaCO_2_ change contribute to its potential impact on CA and neurological complications. The magnitude of these changes is likely to be greatest in the early ECMO period and coincides with the first 24 h following ECMO, representing the most critical period of CA susceptibility to impairment [35]. Although the exact role of hyperocapnia and hypocapnia in CA remains to be determined, there is sufficient evidence to suggest that these ventilatory alterations may impair CA at the time of ECMO transition, predisposing to cerebral injury secondary to changes in MAP and passive CBF.

#### Mechanical Ventilation and Positive End-Expiratory Pressure

Patients with respiratory insufficiency who require mechanical ventilation may already have CA dysfunction prior to transition to ECMO. Though paramount to facilitating gas exchange, positive intrathoracic pressure can restrict central venous drainage, potentially increasing ICP. Previous studies evaluating the relationship between positive end-expiratory pressure (PEEP) and ICP or CPP for various neurological injuries have described mixed results [115–118]. More recently, Boone et al. observed that this relationship is affected by the degree of underlying lung injury [119]. A significant positive relationship between PEEP and both ICP and CPP was found in patients with severe acute lung injury but not in those without severe acute lung injury [119]. Thus, PEEP could potentially be safe in patients with severe brain injury but not in those with severe acute lung injury [119]. Transmitted effects of PEEP on CPP were relatively modest; a 10-cm H_2_O increase in PEEP could potentially decrease CPP by 8.6 mm Hg in these most vulnerable patients [119]. Of important consideration, patients with intact CA would be expected to maintain constant CBF despite changes in CPP. However, in patients with impaired CA, these pressure changes may lead to a pressure-passive decrease or increase in CBF, leading to secondary injury.

Among adults with ECMO-dependent respiratory failure, an increased incidence of neurological complications secondary to large relative changes in carbon dioxide has been identified in adults with a longer duration of mechanical ventilation [114]. This finding may represent longer exposure to hypercapnia [114]; however, potential CA impairment secondary to prolonged exposure to positive pressure ventilation may also be a critical contributor.

#### ECMO Flow Rate

Given the necessity of ECMO support for gas exchange and/or circulation, ECMO flow rates may directly impact cerebral perfusion. Experimental models in lambs and rabbits (3–6.2 kg) have found impaired cerebral perfusion at VA ECMO flow rates ≤ 50 mL/kg/min [86, 120] versus preserved cerebral perfusion at 150 mL/kg/min in both lambs and newborn baboons [85, 86, 121]. Clinical studies have also identified higher VA ECMO flow rates to be associated with increased CBF in infants [51, 122]. This association suggests possible CA impairment, raising concern for an increased risk of cerebral hemorrhage at higher flow rates. This concern was examined in the study by O’Brien et al. in which children who developed cerebral hemorrhage had higher than normal CBF velocities prior to recognition of hemorrhage [65]. However, no difference in flow rate was found between children with increased CBF and those without increased CBF [65].

Higher flow rates may also be associated with a greater degree of hemolysis and increased NO scavenging by cell-free heme. Cerebrovascular injury may arise secondary to hemolysis-induced cerebral vasoconstriction [123], contributing to CA impairment. Patients with sickle cell disease have dynamic CA impairment, though investigations have yet to find an association between CA impairment and the severity of hemolysis [124, 125]. Thus, the implication of ECMO flow rates in CA impairment and cerebral injury remains poorly understood and is impacted by patient characteristics, including clinical deterioration; a higher minimum VA ECMO flow rate during the first 4 h of ECMO has been associated with cerebral desaturation [74]. Decreases in VA ECMO flow rate from baseline have also been associated with cerebral hemorrhage and loss of CA [3, 49]. However, pump flow rates did not correlate with DCS-derived CBF in neonates on VA ECMO [42]. It remains unclear whether changes in ECMO flow rates have the potential to impair CA primarily, or whether changes in ECMO flow may result in cerebral injury solely in patients with preexisting CA impairment.

#### Temperature Management

Targeted temperature management (TTM), the application of various interventions to reach and maintain a specific body temperature, may play a cerebral protective role among patients supported by ECLS [126]. Potential benefits of TTM, specifically induced hypothermia, include reduced inflammation, minimized ischemia–reperfusion injury, increased oxygen delivery, increased systemic vascular resistance, improved gas exchange and protective ventilation, and neuroprotection in patients on VV or VA ECMO [126]. However, there is an absence of data evaluating TTM in VV ECMO [126] and a paucity of inconclusive data evaluating its application in VA ECMO for cardiogenic shock [127]. Comparatively, hypothermic TTM may play a role in the preservation of CA in patients after return of spontaneous circulation (ROSC). Valkov et al. recently demonstrated in an experimental study that CA was preserved during the first 2 h of cardiopulmonary resuscitation if swine were surface cooled to 27 °C [128]. In later phases of hypoperfusion, moderate hypothermia has been shown to decrease the LLA in post-arrest piglets, potentially providing a protective effect on the preservation of CA [129]. Intact CA has also been documented in a cohort of adults successfully resuscitated from out-of-hospital cardiac arrest treated with therapeutic hypothermia [130]. Four recent meta-analyses have been published evaluating hypothermic TTM in ECPR [131–134], and all but one [132] found an improvement in neurological outcomes. Though the role of hypothermic TTM remains to be completely elucidated, hyperthermia may be detrimental in the post-arrest period and has been associated with impaired CA in adults [135, 136]. Because the potential benefits of hypothermic TTM in ECLS are not yet fully known, particularly in pediatric populations, normothermia remains the standard clinical target.

#### Blood Pressure Management

Individualized management of blood pressure is a key emerging application of CA monitoring. During VA ECMO following cardiac arrest, also known as ECPR, Ryu et al. identified an association between an initial MAP < 70 mm Hg and poor neurological outcomes, a finding potentially related to impaired CA [137]. More recently, a single-center retrospective study of 253 adults during ECPR found that an average MAP of 75 mm Hg had the best neurological outcomes [138]; an average MAP outside the range of 60–75 mm Hg resulted in worse neurological outcomes, highlighting the danger of both higher and lower MAPs in the face of potentially impaired CA [138]. These results suggest the existence of an MAP_opt_ that is neuroprotective and preserves CA.

Patient-specific determination of MAP_opt_ and the ULA and LLA using continuous noninvasive CA monitoring provides the possibility of detecting when MAP is outside the “safe zone” in which CA is intact [139]. Emerging evidence suggests MAP_opt_, LLA, and ULA are highly patient specific and dynamic. For example, a cerebral insult may cause the LLA to increase above a patient’s current MAP [18]; this phenomenon is known as right shifting of the CA limit. Timely detection of impaired CA and clinical interventions to increase MAP above the LLA may mitigate secondary injury. Experimental data in newborn lambs suggest a higher MAP_opt_ in VA ECMO compared to VV ECMO [68, 70]. Although these data suggest that a higher MAP may be beneficial in VA ECMO, clinical data have also associated intracranial hemorrhage with higher blood pressure ranges [48].

Recent pilot studies have established the feasibility of monitoring the COx of CA (correlation of NIRS rSO_2_ and MAP) at the bedside in pediatric patients throughout ECMO support [34, 35]. Alteration of the limits of CA were most dynamic during the first 24 h following transition to ECMO in pediatric patients, representing a critical time for CA monitoring and intervention [35]. Johnson et al. also reported significant variability in the MAP ranges associated with symmetrical hemispheric CBF in adults during ECMO (predominantly VA ECMO) [52]. Given this variability, there appears to be value in monitoring CA and identifying patient-specific MAP ranges to titrate blood pressure appropriately in the face of its impairment. This value is supported by the recent finding that the time spent outside of the LLA and ULA, derived using COx, was associated with increased incidence of acute neurological events in pediatric ECMO patients (predominantly VA ECMO) [35]. Of important consideration, risk of CA impairment and neurological injury appears to be highest in the first few hours to days of ECMO [3, 35, 63, 95], a point that is important to consider in the context of future CA research investigating blood pressure management strategies.

Dynamic alterations in MAP limits of CA have also been observed in patients during ECPR. Wide variability in MAP limits were observed in a cohort of seven adults in which intact CA was defined by symmetry of cerebral hemispheric blood flow [52]. In a porcine model of ECPR with an average flow of 40 mL/kg/min, an epinephrine infusion was set to target either a standard or high MAP target (65–75 vs. 80–90 mm Hg) before and after ROSC [140]. CA was monitored using the pressure reactivity index (correlation of invasive ICP and MAP). The higher MAP group had better preservation of CA in early ECPR (i.e., 30-min period before defibrillation and ROSC). However, following defibrillation and ROSC, the higher MAP group experienced subsequent deterioration of cerebral hemodynamics and CA impairment 30 min after ROSC [140]. These results highlight the dynamic nature of CA and caution that CA preservation alone may not be sufficient to predict or mitigate future neurological injury.

Lee et al. evaluated MAP_opt_ via the hemoglobin volume index (correlation between NIRS-derived relative tissue hemoglobin volume and MAP) in 36 pediatric cardiac arrest patients, including 10 who required ECMO; although a significant association was found between the area under the curve of time spent with MAP below individualized MAP_opt_ and change in Pediatric Cerebral Performance score across all patients, no association was observed in ECMO patients alone [50]. This highlights the need for more clinical studies to elucidate the relationship between MAP_opt_ and neurological outcomes during ECMO.

Patient-specific MAP_opt_ monitoring and blood pressure targeting are appealing in various patient populations supported by ECMO; however, additional research is needed to explore optimal determination of MAP limits and whether blood pressure management targeted to MAP_opt_ ultimately improves outcomes in patients supported by ECMO compared to traditional blood pressure management.

## Conclusions

There is increasing evidence that CA is impaired in various forms of ECMO. Although medical advances have contributed to increased availability and reliability of mechanical circulatory support, the incidence of neurological complications in VV and VA ECMO remains strikingly high. Given emerging evidence of impaired CA and its association with poor neurological outcomes, CA may yield new opportunities to optimize cerebral health. At present, there exists heterogeneity among existing studies in the definitions applied and methods used to assess CA and MAP_opt_. There is an urgent need for standardization of CA indices among the international resuscitation community. Consideration for the inclusion of CA and MAP_opt_ in future revisions to Utstein-style guidelines may facilitate this standardization. In the face of dynamic pathophysiological derangements, monitoring and preserving CA may help improve neurological outcomes in ECLS.
